# Short-term intravenous antimicrobial prophylaxis for elective rectal cancer surgery: results of a prospective randomized non-inferiority trial

**DOI:** 10.1007/s00595-013-0695-1

**Published:** 2013-08-29

**Authors:** Keiichiro Ishibashi, Hideyuki Ishida, Kouki Kuwabara, Tomonori Ohsawa, Norimichi Okada, Masaru Yokoyama, Kensuke Kumamoto

**Affiliations:** Department of Digestive Tract and General Surgery, Saitama Medical Center, Saitama Medical University, 1981 Kamoda, Kawagoe, Saitama 350-8550 Japan

**Keywords:** Rectal cancer, Antimicrobial prophylaxis, Surgical site infection

## Abstract

**Purpose:**

To investigate the non-inferiority of postoperative single-dose intravenous antimicrobial prophylaxis to multiple-dose intravenous antimicrobial prophylaxis in terms of the incidence of surgical site infections (SSIs) in patients undergoing elective rectal cancer surgery by a prospective randomized study.

**Methods:**

Patients undergoing elective surgery for rectal cancer were randomized to receive a single intravenous injection of flomoxef (group 1) or five additional doses (group 2) of flomoxef after the surgery. All the patients had received preoperative oral antibiotic prophylaxis (kanamycin and erythromycin) after mechanical cleansing within 24 h prior to surgery, and had received intravenous flomoxef during surgery.

**Results:**

A total of 279 patients (including 139 patients in group 1 and 140 in group 2) were enrolled in the study. The incidence of SSIs was 13.7 % in group 1 and 13.6 % in group 2 (difference [95 % confidence interval]: −0.2 % [−0.9 to 0.7 %]).

**Conclusion:**

The incidence of SSIs was not significantly different in patients undergoing elective rectal surgery who were treated using a single dose of postoperative antibiotics compared to those treated using multiple-dose antibiotics when preoperative mechanical and chemical bowel preparations were employed.

## Introduction

To prevent surgical site infections (SSIs) in patients undergoing colorectal surgery, the guidelines of the Centers for Disease Control and Prevention (CDC) published in 1999 [1] recommend the administration of a brief course of chemical preparations after mechanical bowel cleansing and limited use of intravenous antimicrobial prophylaxis (within 24 h of surgery). The validity of using limited-dose (such as single-dose) intravenous antimicrobial prophylaxis was demonstrated by a meta-analysis that included 17 randomized controlled trials [2]. However, these randomized controlled trials (RCTs) involved subjects undergoing colon surgery alone or a mixture of subjects undergoing colon and rectal surgery, with a lower percentage of those undergoing rectal surgery (range 18.6–55.0 %). Of these trials, only three [3–5] reported the incidence of SSIs in patients undergoing rectal surgery. Although the results indicated that there was no significant difference between single-dose and multiple-dose intravenous antimicrobial prophylaxis in terms of the reduction in the incidence of SSIs, the number of cases in each group was too small, ranging from only 18 to 49 cases.

In comparison to surgery for colon cancer, surgery for rectal cancer involves more extensive procedures, such as abdomino-perineal resection and pelvic exenteration. In addition, lateral lymph node dissection is frequently performed for cases of lower rectal cancer in Japan [6]. Ostomy construction, preoperative radiation and a very low anastomosis are all associated with a prolonged duration of surgery, greater risk of bacterial contamination and a wider dead space [7–10]. Previous reports from Western countries have suggested that the incidence of SSIs might differ between colon and rectal surgery [11, 12]. Two important reports from Japan [13, 14] demonstrated a higher incidence of SSIs following rectal surgery than following colon surgery, even after the inclusion of various types of surgeries in the analyses. Therefore, determining the usefulness of short-term intravenous antimicrobial prophylaxis specifically for reducing the incidence of SSIs in patients undergoing rectal surgery would be of interest.

We carried out a prospective randomized non-inferiority trial to evaluate the usefulness of short-term intravenous antimicrobial prophylaxis combined with preoperative chemical preparation (administration of an antibiotic(s)), in terms of its efficacy for reducing the incidence of SSIs, and to identify the risk factors for SSIs in patients undergoing elective rectal cancer surgery in Japan.

## Patients and methods

This study was conducted with the approval of the ethics committee of Saitama Medical Center, Saitama Medical University. Written informed consent was obtained from each patient.

### Patients

A total of 295 patients underwent elective resectional surgery for rectal cancer, including rectosigmoid cancer, at our institution between January 2003 and September 2011.

### Preoperative and intraoperative procedures related to the development of SSIs

All of the patients were given kanamycin (3 g/day) and erythromycin (2.4 g/day) orally in three divided doses after mechanical bowel cleansing, within 24 h prior to surgery, in accordance with the CDC guidelines [1]. The mechanical bowel preparation consisted of bowel lavage with 2 L of polyethylene glycol or 34 g of magnesium citrate. Thereafter, 1 g of flomoxef (FMOX), a second-generation cephalosporin, was administered by intravenous injection, with an additional dose administered when the duration of surgery exceeded 3 h.

The surgical wounds were covered with surgical towels. A stapled anastomosis was routinely performed for anterior resections. The stump of the Hartmann’s pouch was also closed with a stapler. A hand-sewn anastomosis was made for intersphincteric resections. Irrespective of the type of surgery performed, the abdominal cavity was washed with copious amounts (2–3 L) of saline before closure of the wounds, and a closed-suction drain (BLAKE^®^ silicone drains, Ethicon, Johnson & Johnson, Somerville, NJ, USA) was placed presacrally, brought out through a separate stab wound, and connected to a J-VAC^®^ suction reservoir (Ethicon, Johnson & Johnson, Somerville, NJ, USA). All gloves were changed after the abdominal cavity was washed. After the fascia was approximated with absorbable sutures, the incisional site of the abdominal wall was washed with 200 mL of saline before closure of the skin, which was approximated with a skin stapler. The perineal skin was approximated with 2-0 non-absorbable sutures when abodomino-perineal resection or pelvic exenteration was performed.

### Randomization of the patients

After surgery, the patients were assigned to one of the following two groups using sealed envelopes containing randomized sheets. The patients in group 1 were given only single-dose intravenous antimicrobial prophylaxis 1 h after completion of the surgery, while the patients in group 2 were given an additional 5 doses over 2 consecutive days. Patients less than 20-year old, those with a known allergy to FMOX and those with any infection diagnosed within the previous 2 weeks, were excluded from this study.

### Postoperative follow-up

The incision site was covered with a sterile dressing, which was removed within 48 h of surgery. In principle, the pelvic drain was removed within 5 days of surgery, and the staples were removed on postoperative day 7. The wounds were inspected daily until the patients were discharged from the hospital, and each patient’s wounds were inspected at the outpatient clinic 30 days after surgery. SSIs (incision site infections and organ/space infections) were recorded according to the definitions of the CDC [1]; however, no distinction was made between superficial and deep SSIs, because discrimination between the two was often difficult. Anastomotic dehiscence, which was classified into organ/space infection, was confirmed by clinical and/or radiographic examinations. Remote infection was defined as an infection that occurred at a site other than the surgical site, such as pneumonia, enteritis, urinary tract infection or bloodstream (catheter-related) infection. The distribution of the location of the primary tumor and level of lymph node dissection was determined according to the guidelines of the Japanese Society for Cancer of the Rectum and Anus [15], and the distribution of the pathological stage was determined according to the TNM classification [16].

### Sample size calculation

This trial was designed as a non-inferiority test to detect a 10 % difference in the incidence of SSIs between the two groups with a confidence interval (CI) of 95 % and statistical power of 80 %, assuming that the incidence of SSIs in the multiple-dose intravenous antimicrobial prophylaxis group (group 2) would be 12 %, based on the our previous data on the incidence of SSIs after rectal cancer surgery performed between September 2000 and September 2001. Based on the above, it was calculated that a sample size of 131 would be required in each treatment arm. Then, the required number of patients was set at 140 per group, assuming a 10 % potential dropout rate. [17].

### Statistical analysis

The results in the single-dose prophylaxis group (group 1) were considered not to be inferior to those in the multiple-dose prophylaxis (group 2) if the lower limit of the two-sided 95 % CI for the difference in the incidence of SSIs was above −10 %. The data are expressed as medians and ranges or 95 % CI. For the statistical analyses, a statistical software package (StatView ver. 5.0, Abacus Concepts, Inc, Berkeley, CA, USA) running on a Windows personal computer was used. For the comparison of nominal variables, either the Chi-Square test or Fisher’s exact probability test was used. For the comparison of continuous variables, Mann–Whitney’s *U* test was used. *P* values of <0.05 were considered to denote statistical significance.

## Results

### Eligible patients

**Fig. 1:**
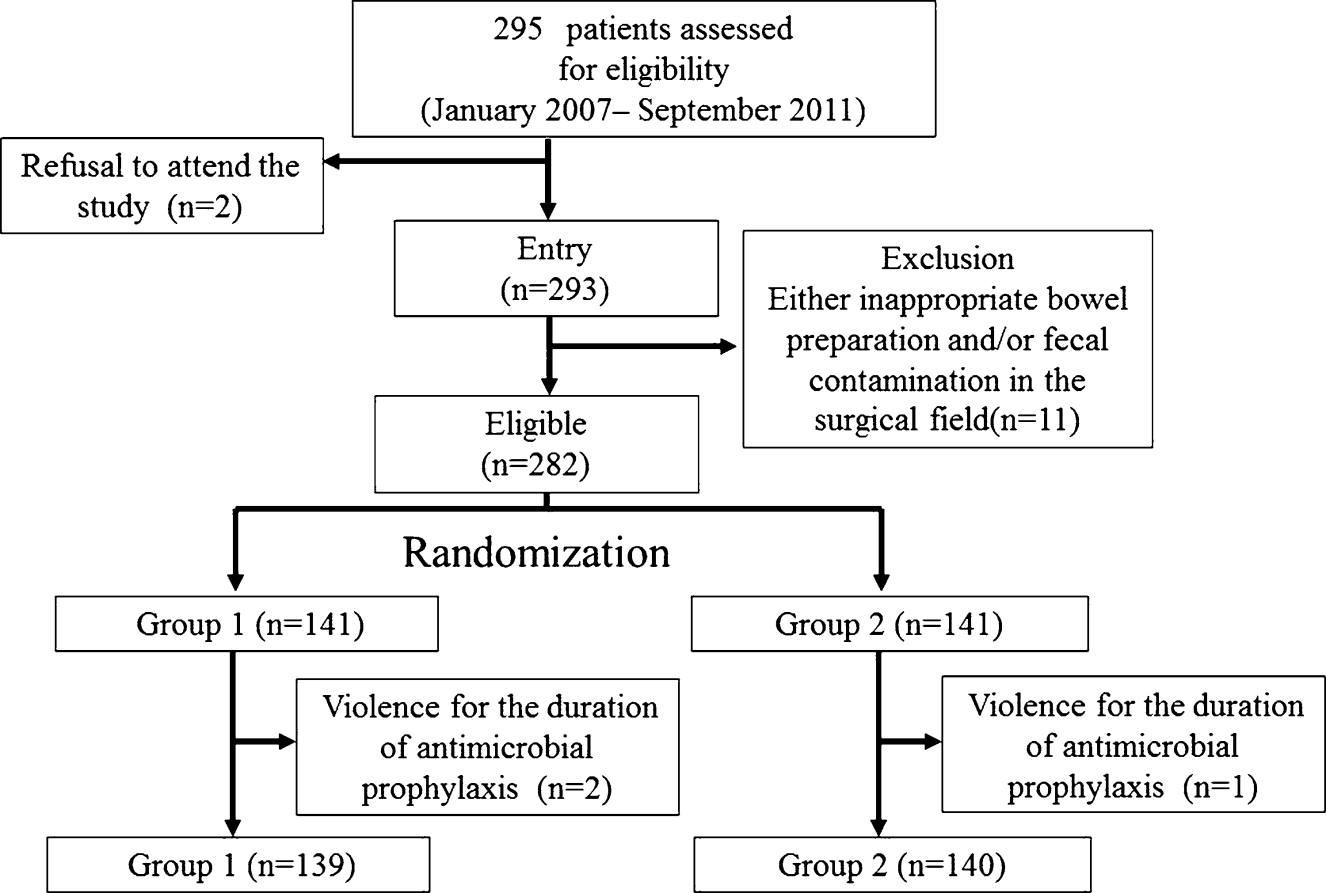
A flow-chart of the randomization of the patients

A flow chart of the randomization of the patients is shown in Fig. 1. Among the 295 patients who underwent elective surgery for rectal cancer during the specified period, a total of 16 patients were excluded, owing to refusal to participate in the study (two patients), inappropriate bowel preparation and/or fecal contamination in the surgical field (11 patients), or protocol violation with respect to the duration of antimicrobial prophylaxis after randomization (three patients, including two from group 1 and one from group 2). Thus, a total of 279 patients (*n* = 139 for group 1 and *n* = 140 for group 2) were finally enrolled in the study.

### Patient characteristics

The two groups did not differ significantly with respect to the sex ratio, age, ASA (American Society of Anesthesiologists physical status) score, BMI (body mass index), prevalence of diabetes mellitus, distribution of the location of the primary tumor, type of surgery, frequency of stoma creation, frequency of lateral lymph node dissection, frequency of combined resection of other organ(s), curative intent of surgery, duration of surgery, blood loss, level of lymph node dissection or distribution of the pathological stage (Table 1).

**Table 1:** Patient characteristics

	Group 1 (*n* = 139)	Group 2 (*n* = 140)	*P* value
Sex (male:female)	88:51	92:48	0.18
Age (years) ^a^	65 (35–90)	65 (33–91)	0.81
ASA^b^ score (I/II:III)	127:12	125:15	0.56
BMI (body mass index)^a^ (kg/m^2^)	22.0 (15.6–32.0)	22.4 (13.2–33.0)	0.57
Diabetes mellitus (present:absent)	16:123	15:125	0.83
Location of the primary tumor			0.18
Rectosigmoid	46	38	
Upper rectum	33	47	
Lower rectum	60	55	
Type of surgery			0.99
Hartmann’s procedure	4	5	
Anterior resection	110	109	
Intersphincteric resection (ISR)	5	6	
Abdomino-perineal resection	18	19	
Pelvic exenteration	2	1	
Total pelvic exenteration	1	1	
Posterior (anterior) pelvic exenteration	1	1	
Stoma creation (%)	44 (31.7%)	47 (33.6%)	0.73
Colostomy (%)	25 (18.0%)	24 (17.1%)	
Ileosomy (%)	19 (13.7%)	23 (16.4%)	
Lateral lymph node dissection performed (%)	39 (28.1%)	30 (21.4%)	0.20
Combined resection of other organ(s)	19 (13.7%)	31 (22.9%)	0.07
Liver	4	8	
Small intestine or colon	1	2	
Urinary bladder	4	6	
Others	11	17	
Curative intent of surgery^c^			0.12
R0	120	129	
R1/R2	19	11	
Duration of surgery (min)^a^	187 (83–670)	185 (80–495)	0.75
Blood loss (g)^a^	260 (10–5850)	310 (10–3120)	0.39
Lymph node dissection^d^			0.93
D0/D1	6	6	
D2	18	16	
D3	115	118	
pTNM stage^c^			0.11
Stage 0/I/II	65	79	
Stage III/IV	74	61	

^a^Median (range)

^b^American Society of Anesthesiologists physical status

^c^According to the 7th TNM Classification

^d^According to the Japaneses Classification of Colorectal Carcinoma

### SSIs

The incidence of incision site infections was 5.0 % (seven patients) in group 1 and 7.1 % (10 patients) in group 2. All the incision site infections were considered to be superficial incision site infections. The incidence of organ/space infections was 10.8 % (15 patients) in group 1 and 8.6 % (12 patients) in group 2. Of the 27 organ/space infections, 12 (five in group 1 and seven in group 2) were related to anastomotic dehiscence. Three patients in group 1 and three patients in group 2 developed both incision site and organ/space infections. Therefore, the overall incidence of SSIs was 13.7 % (19 patients) in group 1 and 13.6 % (19 patients) in group 2. The difference [95 % CI] in the incidence of SSIs between the two groups was −0.2 % [−0.9 to 0.7 %]. Because the lower limit of the two-sided 95 % CI was above −10 %, the outcome in terms of the incidence of SSI in group 1 was considered to be non-inferior to that in group 2 (Table 2).

**Table 2:** Surgical site infections

	Group 1 (*n* = 139)	Group 2 (*n* = 140)	Difference (95 %CI)
Overall surgical site infections	19 (13.7 %)	19 (13.6 %)	−0.2 % (−0.9 to 0.7 %)
Incisional site infections	7 (5.0 %)	10 (7.1 %)	
Organ/space infections	15 (10.8 %)	12 (8.6 %)	

### Subset analysis for SSIs

A subset analysis was performed based on the types of surgery, namely, surgeries associated with a wide pelvic dead space (abdomino-pelvic resection and pelvic exenteration) vs. other surgeries, including anterior resection, intersphincteric resection, and Hartmann’s procedure. In terms of the surgeries with a wide pelvic dead space, the overall incidence of SSIs (40.0 vs. 30.0 %, *P* = 0.51), incision site infections (5.0 vs. 20.0 %, *P* = 0.15) and organ/space infections (35.0 vs. 15.0 %, *P* = 0.14) did not differ significantly between group 1 and group 2. In terms of the surgeries, including anterior resection, intersphincteric resection and Hartmann’s procedure, the overall incidence of SSIs (9.2 vs. 10.8 %, *P* = 0.68), incision site infections (5.0 vs. 5.0 %, *P* > 0.99) and organ/space infections (6.7 vs. 7.5 %, *P* = 0.82) also did not significantly among between the groups (Table 3).

**Table 3:** Surgical site infections according to the type of surgery

	Overall surgical site infections			Incisional site infections			Organ/space infections		
	Group 1 (*n* = 139)	Group 2 (*n* = 140)	*P* value	Group 1 (*n* = 139)	Group 2 (*n* = 140)	*P* value	Group 1 (*n* = 139)	Group 2 (*n* = 140)	*P* value
APR or PE (*n* = 40)	8/20 (40.0 %)	6/20 (30.0 %)	0.51	1/20 (5.0 %)	4/20 (20.0 %)	0.15	7/20 (35.0 %)	3/20 (15.0 %)	0.14
AR, ISR or HA (*n* = 239)	11/119 (9.2 %)	13/120 (10.8 %)	0.68	6/119 (5.0 %)	6/120 (5.0 %)	>0.99	8/119 (6.7 %)	9/120 (7.5 %)	0.82

*AR* anterior resection, *ISR* intersphincteric resection, *HA* Hartmann’s procedure, *APR* abdomino-perineal resection, *PE* total (posterior or anterior) pelvic exenteration

### Remote infections

A remote infection was detected in nine (6.5 %) and 15 patients (10.7 %) in groups 1 and 2, respectively; there was no significant difference in the incidence of remote infection between the groups (*P* = 0.21) (Table 4). Among the patients who developed enterocolitis, *Clostridium difficile* toxin A was detected in one patient from Group 1. This patient was treated with vancomycin and recovered without further complications.

**Table 4:** Remote site infections

	Group 1 (*n* = 139)	Group 2 (*n* = 140)	*P* value
Remote site incisions	9 (6.5 %)	15 (10.7 %)	0.21
Pneumonia	0	3	
Enterocolitis	3	4	
Urinary tract infections	4	6	
Bloodstream infections	2	2	

## Discussion

This study clearly showed that, in patients administered a brief course of a chemical preparation after mechanical bowel cleansing prior to rectal cancer surgery, postoperative single-dose intravenous antimicrobial prophylaxis was non-inferior to multiple-dose prophylaxis in terms of the subsequent incidence of SSIs. Importantly, it appears that, irrespective of the type of rectal cancer surgery performed, selection between postoperative single-dose and multiple-dose intravenous antimicrobial prophylaxis in the immediate postoperative period may have little impact on the risk of the development of SSIs.

Even though this study was performed at a single institution, we believe that it had several distinct merits that enhanced its quality. For example, there were no inter-hospital variations, and the surgical procedures and pre-, intra- and postoperative management protocols related to SSIs were well standardized. To the best of our knowledge, this is the first randomized study comparing postoperative single-dose vs. multiple-dose intravenous antimicrobial prophylaxis following a course of oral antibiotics and mechanical bowel preparation prior to elective rectal cancer surgery, while Suzuki et al. [18] and our own group [19] have previously reported the non-inferiority of single-dose to multiple-dose intravenous antimicrobial prophylaxis in elective colon cancer surgery performed after preoperative chemical and mechanical preparation.

The National Nosocomial Infections Surveillance (NNIS) system categorizes all colorectal surgeries into the same “COLO” group, and the incidence of SSIs within this group is stratified according to the NNIS risk index calculated based on the following three factors: the ASA score, wound classification and duration of operation (with 3 h set as the cut-off point) [20]. The NNIS risk index has been criticized as being unsuitable for risk evaluation in patients undergoing elective colorectal surgery, because most patients undergoing colorectal surgery have an ASA score of 1 or 2 and a clean-contaminated wound [10, 21, 22]. In terms of the incidence of incisional site infections, a retrospective analysis by Konishi et al. [13] demonstrated that the incidence in patients undergoing elective rectal surgery was 18.0 %, nearly twice as high as the incidence of 9.4 % in patients undergoing elective colon surgery, although they did not evaluate organ/space infections. The Japanese Nosocomial Infection Surveillance (JNIS) system analyzed the incidence of SSIs after elective and emergency colon surgery and rectal surgery separately in 2009, and reported that the incidence of SSIs was 12.7 % after colon surgery and 16.3 % after rectal surgery [23]. It was therefore unclear whether the same perioperative management strategies, including the duration of intravenous antimicrobial prophylaxis, would be useful for both rectal and colon surgery.

In our present study, none of the patients had received preoperative chemoradiotherapy. In Western countries, chemoradiation prior to surgery is a standard treatment for locally advanced rectal cancer [24]. In Japan, surgery without neoadjuvant treatment, occasionally with lateral lymph node dissection, is the standard treatment for lower rectal cancer [6], even though (chemo) radiation for both upper and lower rectal cancers is performed in some institutions. A large randomized controlled study comparing total mesorectal excision (TME) with and without preoperative radiation for rectal cancer demonstrated the absence of any significant difference in the overall incidence of SSIs between the two groups [25]. Notably, according to some previous studies, TME, including both low anterior resection and abdomino-perineal resection, was associated with a high incidence of SSIs of 27.7 and 31.6 % for the two aforementioned procedures, respectively [21], even in the absence of preoperative radiation. Nevertheless, a short course of intravenous antimicrobial prophylaxis in patients receiving preoperative radiation deserves investigation in the future.

There were two important randomized trials [26, 27] from Japan that have compared the risks of SSIs following single-dose intravenous antimicrobial prophylaxis [26] and preoperative chemical preparation [27] in patients undergoing elective colorectal surgery. Fujita et al. [26] reported that single-dose intravenous prophylaxis was associated with a >3-fold increase in the overall incidence of SSIs (14.3 vs. 4.3 %) compared to triple-dose intravenous antimicrobial prophylaxis in patients undergoing elective colorectal cancer surgery who had undergone mechanical cleansing, but not chemical preparation, preoperatively. They did not analyze the results separately in patients undergoing colon cancer surgery and rectal cancer surgery. Kobayashi et al. [27] reported that, in patients administered intravenous antimicrobial prophylaxis until postoperative day 3, the use of the chemical preparation was not associated with a reduction in the overall incidence of SSIs; however, in a subset analysis, the use of the chemical preparation was associated with a significantly reduced incidence of SSIs in patients undergoing abdomino-perineal resection (58.8 vs. 11.1 %). This result was not consistent with our results demonstrating no significant differences in the incidence of SSIs after abdomino-perineal resection/pelvic exenteration between patients receiving single-dose and those receiving multiple-dose intravenous antimicrobial prophylaxis postoperatively (*P* = 0.74). These findings suggest that the risk of SSIs after abdomino-perineal resection might be influenced by the choice of chemical preparation and/or the duration of intravenous antimicrobial prophylaxis.

It has been reported that preoperative oral antibiotic use can induce *Clostridium difficile*-related colitis [28]. In our study, we detected *Clostridium difficile* toxin A in only one patient, who recovered with only conservative therapy. Because we did not conduct routine testing for *Clostridium difficile*, the exact incidence of “subclinical” *Clostridium difficile*-related infection in our patients remains unknown. Further studies are warranted to clarify this issue; however, a recent retrospective multicenter study demonstrated that chemical preparation did not increase the risk of *Clostridium difficile* infection in patients undergoing elective colon surgery [29].

In summary, our results suggest that the incidence of SSIs in patients undergoing elective rectal surgery following single-dose postoperative use of antibiotics was not significantly different from that of those undergoing multiple-dose treatments as long as preoperative mechanical and chemical bowel preparations were employed. Therefore, the administration of a single-dose of postoperative antibiotics is a valid measure to prevent postoperative surgical site infections.
